# Unanticipated efficacy of SARS-CoV-2 vaccination in older adults

**DOI:** 10.1186/s12979-021-00219-y

**Published:** 2021-02-17

**Authors:** Graham Pawelec, Janet McElhaney

**Affiliations:** 1 Department of Immunology, University of Tübingen, Tübingen, Germany; 2 Health Sciences North Research Institute, Sudbury, Ontario Canada

**Keywords:** SARS-CoV-2, COVID-19, Immunosenescence, Vaccination, Paradigm shift

## Abstract

The rapidity with which vaccines against COVID-19 have been developed and tested is unprecedented. As classically the case with randomized clinical trials, many studies excluded older adults. However, given the early realisation that senior citizens were most highly susceptible to COVID, older individuals have been included in licensing trials under these unusual conditions. The recently published results from the Comirnaty Vaccine (BNT162b) trial unexpectedly documented that vaccine efficacy was equally exceptionally high in older and younger adults. These extremely encouraging trial results with a neoantigen vaccine may suggest the beginning of a paradigm shift in our view of the impact of immunosenescence on vaccination against novel infectious diseases.

## Introduction

Immunosenescence causes increased susceptibility and severity of infectious disease, poor responses to vaccination, and increased autoimmunity and cancer – or so the received wisdom as reflected in innumerable publications has it. The current paradigm as recently formulated during a consensus-seeking workshop [1] is that age-associated alterations to hematopoiesis result in a skewed output of immune cells to the periphery, with fewer lymphocytes and more myeloid cells. This paucity of T-cell precursors together with developmentally-programmed thymic involution results in markedly decreased generation of functional naïve T cells and contributes to a reduced T cell receptor repertoire, thus compromising the ability of the individual to respond to neoantigens. Changes to myeloid antigen-presenting cells and altered lymph node architecture further contribute to decreased capacity to respond to new challenges. At the same time, changes to B cells further impact humoral immunity, and accumulations of senescent cells contribute indirectly to negative effects by suppressive activity and directly by taking up “immunological space”. All this takes place on a background of undefined changes to the overall systemic environment – “old blood”. According to this paradigm, older adults are predicted to respond poorly or not at all to neoantigens, providing a basis for the near-universal observation that infectious agents newly-introduced into previously-unexposed populations have dramatic consequences. Hence the prediction that immunosenescence is one reason why older adults are so much more susceptible to COVID-19 than the young, and the expectation that the SARS-CoV-2 spike antigens contained in the current COVID vaccines would trigger less effective immunity in older adults, as we argued in an earlier Editorial published last April, 2020. We stated “we consider it unlikely that a conventional vaccine based on young adult responses will be highly effective in COVID prophylaxis for older adults”. Fortunately, but for many investigators unexpectedly, this does not appear to be the case with the current RNA vaccines for which preliminary data on efficacy do not implicate a negative effect of age, according to detailed data published 31st December, 2020 [2]. Does this represent the beginning of a paradigm shift [3] in our understanding of the impact of immunosenescence on responses to neoantigens?

## Efficacy of the SARS-CoV-2 spike RNA vaccine BNT162b in older adults

Based on their lipid nanoparticle RNA vaccine platform, honed for stimulating T cell responses to neoantigens in melanoma patients [4], the BioNtech company developed and tested two COVID-19 vaccine candidates containing a nucleoside-modified mRNA encoding the entire viral spike glycoprotein of SARS-CoV-2. The lipid for delivery is ((4-hydroxybutyl)azanediyl)bis(hexane-6,1-diyl)bis(2-hexyldecanoate), and the vaccine also contains 2-[(polyethylene glycol)-2000]-N,N-ditetradecylacetamide, 1,2-distearoyl-sn-glycero-3-phosphocholine, and cholesterol), potassium chloride, monobasic potassium phosphate, sodium chloride, dibasic sodium phosphate dihydrate, and sucrose. Based on safety and efficacy data [5], one of these, designated BNT162b, was selected for phase 2/3 testing. This RCT performed by Pfizer with vaccine BNT162b comprised 43,448 participants at least 16 years of age of whom 21,720 received two doses of the vaccine 3 weeks apart, and 21,728 the placebo. One readout for efficacy was the occurrence of symptomatic laboratory-confirmed COVID-19 before 7 days after the second dose in the age-stratified analysis. This was recorded in 162 participants who received placebo but in only 8 who were vaccinated, an unprecedently high efficacy. Breaking down the participants by age revealed that of 3,848 vaccine recipients ≥ 65 years of age only one became infected whereas 19 of 3,880 placebo recipients tested positive. This translates to an estimated efficacy of 94.7 %, compared with 95.6 % in 16–55 year-olds and 93.7 % in those aged 55–65. Nowadays, 65 is no longer considered particularly old, so this trial also included 1,559 participants over 75. Strikingly, none of the 774 vaccinated individuals tested positive, whereas 5 controls did. Given the greater susceptibility to COVID-19 of men than women, and those of certain ethnicities relative to Caucasians, of it is also important to note that the degree of protection was identical in men and women, and did not differ according to ethnicity. These data are thus highly encouraging, but unexpected in the light of the view of immunosenescence outlined in the Introduction. With the ongoing delivery of the BNT162b vaccine prioritizing older adults in most countries, especially those in residential care homes, there should soon be an answer to the question of whether protection is equally good in all age groups in a real-world setting.

## What might explain the unanticipated efficacy of the SARS-CoV-2 RNA vaccine BNT162b in older adults?

First, one must ask how robust are the data from the phase 2/3 Pfizer/BioNtech trial? As the authors point out, the trial was not powered for subgroup analysis, but nonetheless the data do seem very convincing, as reported above. Second, people over 75 might have been less exposed to the virus (1.1 % of the young participants in the placebo group tested positive whereas the older participants were 0.4–0.6 % positive); however, it is hard to believe that this would have made a great difference to the results in the vaccinated group. Third, efficacy was estimated only very early on, and follow-up might reveal less protection at later times, possibly due to individual variation in viral load and control, or re-exposure to the same or different viral variants at a later time point. Fourth, efficacy as measured in controlled RCTs does not necessarily translate to efficacy in the real world. Finally, the placebo was not vehicle alone - only saline; thus an adjuvant effect of vehicle alone cannot be excluded, but this does not affect the fact that individuals who received the vaccine suffered far less infection than those who did not.

Given that we can accept that the BNT162b vaccine truly will have equally high efficiency in adults > 65 or even 75 years-of-age, how might these results be reconciled with the expectation that immunosenescence results in compromised responses to vaccination in older adults, also introducing the crucial question of whether vaccine responses in general are really reduced in older people? The answer to the latter question is easy: a qualified no, they are not. There are precedents for vaccines working just as well in older adults as in younger, particularly the Shingrix vaccine for VZV [6]. However, there is a major crucial difference between a vaccine that boosts a pre-existent immune memory response (Shingrix) and one that must activate naïve immune cells against neoantigens (BNT162b). In the former case, immune cells have been retained within the adaptive immune system whereas in the latter, the paucity of naïve cells at old age may result in holes in the antigen receptor repertoire, compromised antigen processing and presentation capacity and many other possible reasons as discussed above. It is therefore less unexpected that the former type of vaccine should be effective in older adults, but puzzling that the latter should be. These considerations beg several questions, including whether responses to SARS-CoV-2 are truly limited to responses against neoantigens. A significant degree of immune cross-reactivity between the spike protein of SARS-CoV-2 and earlier Coronaviruses such as those causing some common colds is known to exist both at the T and B cell levels, in a significant fraction of the population [7–9] - but to explain the exceptionally high efficacy of BNT162b in older adults one would have to postulate their existence in 100 % of older individuals together with an effective boosting effect of the vaccine. While this has not been specifically tested, for example Braun et al., Mateus et al. and Grifoni et al. examined unexposed donors up to the age of 64 years [7] and 66 years [8, 9], it is conceivable but does seem a priori unlikely. The same applies to the presence of antibodies in unexposed individuals, tested up to the age of 76 (but only in two donors of this age) by Ng et al. [10]. In all instances, it is in any case unknown whether the presence of such cross-reactive T cells and/or antibodies confers resistance to infection or disease, or impacts responses to vaccination, or even hinders protection.

A prime example of the much-studied perceived poorer responses to vaccines by older adults concerns seasonal influenza vaccination. Responses to these vaccines are universally accepted as being worse in older adults but is this really due to immunosenescence? The loss of protection has largely been attributed to diminished antibody responses to seasonal inactivated influenza vaccines with little attention paid to the T cell responses that are needed to protect from severe disease. It is now recognized that the decline in the antibody response to vaccination in older adults may rather be an effect of repeated annual vaccination and not aging per se [11, 12]. Surprisingly, and counter-intuitively, increased frailty in older adults has been associated with an enhanced antibody response to influenza vaccination [13], but this does not necessarily translate to improved protection. When adjusted for the effects of frailty, influenza vaccine effectiveness for the prevention of hospitalization (i.e., severe disease) in older adults is similar to that of young adults [14]. However, the major limitation of inactivated influenza vaccines is that they are poor stimulators of the cytolytic T cell response needed to clear the virus from infected lung tissue. This is in sharp contrast to the SARS-CoV-2 spike RNA vaccine BNT162b, which has been shown to be a potent stimulator of both CD4 (T helper Type 1) and CD8 (cytolytic) T cell responses [5, 15] and translates to improved protection against severe disease [2]. The impact of increasing frailty on the effectiveness of these RNA vaccines remains to be specifically established when they are given to the highest risk group for severe complications of COVID-19 – those frail older adults living in congregated care settings.

As argued in the consensus paper noted above [1] our knowledge of age-associated immune dysfunction and its clinical consequences specifically in people is limited, and relies to a great extent on extrapolation from animal models, especially mice - which are known to be quite different from humans [16]. Clearly, hematopoiesis may be so severely affected in the oldest old that it becomes essentially monoclonal, but what this means for immune status is not clear [17]. Similarly, although age-associated thymic involution drastically reduces the output of naïve T cells and several investigators have concluded that this can lead to holes in the antigen receptor repertoire [18–22], it has also been argued that the resulting repertoire reduction is unlikely to have dramatic clinical consequences due to peripheral compensatory mechanisms [23, 24]. Moreover, most data on antigen repertoire status in humans derive from analyses of peripheral blood and do not encompass potential reservoirs of naïve cells in other tissues, especially lymph nodes [25]. However, there is also some evidence that lymph node architecture is altered in older adults and might compromise their function [26] but this also requires further investigation [27].

## Conclusions

The high efficacy of the SARS-CoV-2 spike RNA vaccine BNT162b in older adults falsifies the hypothesis that immunosenescence due to a composite of age-associated changes to hematopoiesis, antigen receptor repertoire shrinkage, priming against neoantigens and other factors implicated in immune dysregulation necessarily compromises the generation of protective anti-viral immunity.

## Data Availability

Not applicable.
